# Why You Cannot Rank First: Modifications for Benchmarking Six-Degree-of-Freedom Visual Localization Algorithms

**DOI:** 10.3390/s23239580

**Published:** 2023-12-02

**Authors:** Sheng Han, Wei Gao, Zhanyi Hu

**Affiliations:** 1 School of Artificial Intelligence, University of Chinese Academy of Sciences, Beijing 100049, China; sheng.han@nlpr.ia.ac.cn (S.H.); huzy@nlpr.ia.ac.cn (Z.H.); 2 Institute of Automation, Chinese Academy of Sciences, Beijing 100190, China

**Keywords:** visual localization, benchmark enhancement, pose compensation, sequential interpolation, ties resolution

## Abstract

Robust and precise visual localization over extended periods of time poses a formidable challenge in the current domain of spatial vision. The primary difficulty lies in effectively addressing significant variations in appearance caused by seasonal changes (summer, winter, spring, autumn) and diverse lighting conditions (dawn, day, sunset, night). With the rapid development of related technologies, more and more relevant datasets have emerged, which has also promoted the progress of 6-DOF visual localization in both directions of autonomous vehicles and handheld devices.This manuscript endeavors to rectify the existing limitations of the current public benchmark for long-term visual localization, especially in the part on the autonomous vehicle challenge. Taking into account that autonomous vehicle datasets are primarily captured by multi-camera rigs with fixed extrinsic camera calibration and consist of serialized image sequences, we present several proposed modifications designed to enhance the rationality and comprehensiveness of the evaluation algorithm. We advocate for standardized preprocessing procedures to minimize the possibility of human intervention influencing evaluation results. These procedures involve aligning the positions of multiple cameras on the vehicle with a predetermined canonical reference system, replacing the individual camera positions with uniform vehicle poses, and incorporating sequence information to compensate for any failed localized poses. These steps are crucial in ensuring a just and accurate evaluation of algorithmic performance. Lastly, we introduce a novel indicator to resolve potential ties in the Schulze ranking among submitted methods. The inadequacies highlighted in this study are substantiated through simulations and actual experiments, which unequivocally demonstrate the necessity and effectiveness of our proposed amendments.

## 1. Introduction

Estimating the Six-Degree-of-Freedom (6-DOF) pose of robot devices with respect to a known 3D model of the scene is a key technology in spacial vision. It serves as the basis for various applications in 3D scene, including augmented reality [1], visual navigation, SLAM [2], and autonomous vehicles. With the advancement of extensive research, the prevalent techniques for visual localization can be broadly categorized into three distinct groups, image-based methods [3,4,5], structure-based methods [6,7,8,9], and learning-based methods [10,11,12]. All the aforementioned techniques require establishing the connections between the query image and the pre-existing 3D model, thereby highlighting the significance of data association as a pivotal phase in the localization process. The most desirable scenario would entail capturing both the query images and the database images used for constructing the 3D model under precisely identical conditions. However, it is important to acknowledge that this level of congruence may not always align with the practical application requirements of visual localization technology. In a broader context, addressing the disparities in visual attributes between the query and database images poses a significant obstacle for localization algorithms. Advanced algorithms are expected to exhibit exceptional accuracy and robustness, particularly in complex scenarios characterized by substantial fluctuations in lighting conditions, weather patterns, or seasonal variations.

To achieve the aforementioned objectives, researchers have experimented with various techniques to minimize the fluctuation in feature expression while still maintaining the integrity of local image features. These methods include introducing more robust learning-based features [13,14,15], incorporating high-level image semantics [16,17,18], or translating the query and database images into a unified scene [19,20].

In order to impartially assess the localization capabilities of current methods and encourage further research in this field, the long-term visual localization benchmark [21] is proposed for evaluating the effectiveness of 6-DOF visual localization under dynamic conditions. Given the significance of robust visual localization algorithms in the domains of robotics and computer vision, this benchmark has emerged as the primary platform for evaluating such algorithms in the long term. Moreover, it has been adopted as the official evaluation method for numerous international workshops, further establishing its credibility and importance.

While the emergence of the benchmark offers a convenient platform for comparing various algorithms, it is crucial to acknowledge, based on our extensive experience with its usage, that current evaluation systems may possess potential limitations for improvement.

To be specific, firstly, the existing benchmark evaluation method is reasonable and accurate in principle, which can accurately reflect the localization success rate of each camera, but it has an important premise; that is, the evaluated localization results are directly from the output of the visual localization algorithm, original and unmodified. This is natural for datasets captured by hand-held cameras, but for datasets captured by one or more cameras mounted on the vehicle, the extrinsic camera calibration information and the sequence relationship between the images are easily obtained from the dataset, and the existing benchmark cannot determine whether the submitted location results are original, which leaves room for introducing human intervention.

Secondly, the ranking method used by the existing benchmark is a Condorcet method [22], which means that as long as one candidate is ranked higher on most ballots than another candidate, it beats that candidate. In the context of the localization benchmark, because some datasets have a limited number of conditions measured, there may be tied ranking results for the same number of leading conditions. In spite of a strict partial order, the leaderboard lacks a basis for distinguishing the submitted results of tied rankings, which is especially important for the top positions.

In order to establish a research environment that is equitable and conducive to the advancement of relevant fields, in this work, we propose several modifications to improve the two limitations mentioned above.

As for the evaluation method, we advocate a unified process for preprocessing the submitted localization results and then evaluate the accuracy of the processed localization results. The preprocessing involves two main components. Firstly, for datasets captured by multi-camera rigs, the localization results of multiple cameras are aligned with a predetermined canonical reference system, and the resulting unified vehicle poses are utilized for evaluation, replacing the multiple individual camera poses. Secondly, for datasets containing sequence information, any failed localized poses are supplemented by interpolating with several successful localized poses associated with them.

For the ranking method, we introduce a novel index designed specifically for visual localization algorithms, which is used to provide the basis for resolving potential ties in the existing ranking methods, considering the accuracy and robustness of the evaluated algorithms comprehensively. In view of the above, we believe that addressing these limitations can greatly enhance the effectiveness and overall acceptance of the benchmark.

In general, our contributions can be summarized as follows:

- Through rigorous experimentation, we identify two limitations in the existing public benchmark for long-term visual localization evaluation: the inability of the evaluation method to eliminate human intervention, and the lack of judging basis for ranking methods when faced with tied ranking results. These limitations have the potential to hinder the accurate measurement of the localization capabilities of the methods submitted.
- In light of the limitations, we propose a unified process of preprocessing to the current evaluation method employed for localizing methods conducted on multi-camera datasets. Without compromising the practical significance of the autonomous vehicle localization problem, it provides another dimension for the dataset evaluation, which improves the fairness and reliability of the existing benchmark.
- We enhance the current ranking methodology to assist the benchmark in mitigating any potential confusion that may arise from deadlock scenarios when there is a tie between candidates during the ranking calculation process.

The rest of the paper is organized as follows. Section 2 discusses the general framework of existing public benchmarks for visual localization, some relevant typical datasets, and the ranking methods commonly used. Section 3 theoretically explains our proposed modifications, which are divided into two parts: evaluation method modification and ranking method modification. Section 4 is the main part of the paper, in which we conduct a series of experiments, including a simulation experiment for evaluation, an actual experiment for evaluation, and an experiment for ranking, to verify the necessity of our proposed modifications and their impact on benchmark evaluation results. Section 5 presents the findings of each experiment discussed in the preceding section, along with an analysis of their efficacy and the resulting experimental conclusions. In Section 6, we discuss the necessity of our proposed modifications and the far-reaching implications they could have. We also point out the shortcomings of our modifications and give the future research direction. In the final section, we give our conclusion and summarize the main content of the article.

## 2. Related Works

### 2.1. Localization Benchmarks

The benchmark datasets for long-term visual localization ought to encompass query images captured under distinct conditions compared to the database images. This facilitates the analysis of the influence of varying conditions on the accuracy of localization. Nevertheless, it presents challenges in terms of feature matching for the generation of reliable ground truth.

This presents considerable challenges for benchmarking 6-DOF visual localization in changing conditions. On the one hand, datasets with varying environmental conditions [4,12,23,24] present challenges for feature matching, hindering the ability to provide 3D models and 6-DOF ground truth poses, and limiting their use to image-based place recognition. On the other hand, some datasets used for visual localization [6,10,25,26,27,28,29,30,31] are limited by their reliance on feature matching and cannot incorporate images with substantial environmental condition variations.

Recently, Sattler et al. [21] developed a comprehensive benchmark that encompasses ground truth and metrics for long-term visual localization. This benchmark covers localization from both single and multiple images, as well as sequences. The authors diligently annotated matches between images captured under varying conditions to ensure the accuracy and validity of the ground truth.

The benchmark evaluates the localization ability of a method by measuring its pose accuracy by the deviation between the estimated and the ground truth pose. The pose accuracy is determined by position error and orientation error. The position error is evaluated by the Euclidean distance as:

(1) $$\begin{array}{c} {∥C_{est}-C_{gt}∥}_{2}, \end{array}$$

where $C_{est}$ means the estimated query position and $C_{gt}$ means the ground truth position. The absolute orientation error |α| is computed from:

(2) $$\begin{array}{c} 2cos\left(\left|\alpha \right|\right)=trace\left(R_{gt}^{-1}R_{est}\right)-1, \end{array}$$

where $R_{gt}$ and $R_{est}$ represent the estimated and the ground truth camera rotation matrix, respectively.

### 2.2. Benchmark Datasets

The datasets included in the benchmark can be divided into two categories. They differ by how the images are captured: for the first category, images are captured by one or more cameras mounted on a vehicle, and the camera(s) will mostly undergo movement in a plane. Typical representatives are CMU-Seasons dataset [21,32] and RobotCar Seasons dataset [21,33]. For the second category, images are captured with hand-held cameras and thus can undergo arbitrary 6-DOF motion, with Aachen Day-Night dataset [21,29] and InLoc dataset [21,34,35] as typical representatives.

**Figure 1:**
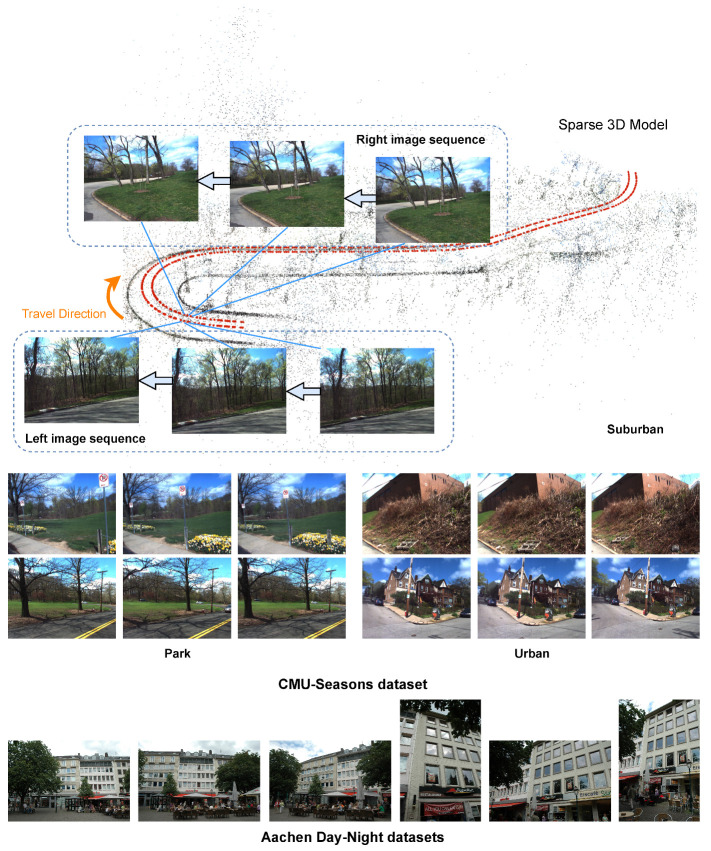
Comparison for example images in CMU Seasons dataset (**top**) and Aachen Day-Night datasets (**bottom**). The CMU Seasons dataset comprises three distinct types of sceneries: Suburban (**top**), Park (**lower left**), and Urban (**lower right**). The image sequences for each of these sceneries are depicted in the accompanying figure. The top three images for each scenery are captured by the right camera, while the bottom three images are captured by the left camera. Additionally, the six images below serve as examples from the Aachen Day-Night datasets and are labeled with consecutive numbers.

The CMU-Seasons dataset used in the benchmark is a subset of the CMU Visual localization dataset by Badino et al. [36]. It showcases various scenes such as urban, suburban, and park scenes in the area of Pittsburgh, USA. The images used for reference and query were taken by a rig of two front-facing cameras mounted on a car, pointing to the left and right sides of the vehicle at an angle of 45 degrees. These images were captured over a period of 1 year. All images were captured in sequences [21]. The CMU Seasons dataset is designed to simulate an autonomous driving scenario, where it is necessary to localize images taken under varying seasonal conditions against a (possibly outdated) reference scene representation. As shown in Figure 1, we sample a set of sequence images from each of the three scenes, and show the correspondence between the images and the sparse 3D model in the Suburban scene. As can be seen from the images, the datasets captured by autonomous vehicles often have a clear back and forth correlation, with relatively little change in perspective.

The RobotCar Seasons dataset is another typical collection of images captured by a camera rig mounted on a vehicle. It is based on a subset of the Oxford RobotCar dataset [33], depicts the city of Oxford, UK. The reference and query images were captured by three synchronized cameras mounted to the left, rear, and right of the car, respectively. Similarly, all images were recorded in sequences [21]. Compared with the CMU-Seasons dataset, it contains additional images taken at night scenes.

The Aachen Day-Night datasets are based on the original Aachen dataset [29], depicts the old inner city of Aachen, Germany. All its database and query images are taken with hand-held cameras. It also includes both daytime and nighttime images, but only daytime captured images are used to construct reference scene representations [21]. In Figure 1, we sample six images labeled with consecutive numbers from the dataset. It can be clearly observed that despite the similarity in shooting positions, there is no notable correlation between the adjacent images in this dataset, and the angle of view changes more significantly.

### 2.3. Ranking Methods

The ranking of the benchmark is performed using the Schultze method from [22]. It is a single-winner election method developed in 1997 by Markus Schulze for internal elections and referendums which satisfies, e.g., resolvability, Condorcet, Pareto, reversal symmetry, monotonicity, and independence of clones.

Due to its numerous positive axiomatic attributes, the Schultze method is extensively utilized in database evaluation. A notable feature is that the Schulze method is a Condorcet method. This indicates that if a candidate is preferred by a majority over every other candidate in pairwise comparisons, then that candidate will emerge as the victor. This characteristic renders the Schultze method highly interpretable and compelling.

However, in certain circumstances, a scenario may occur commonly known as the Condorcet cycle, in which there exists no Condorcet winner. This situation can be likened to a tie, as each candidate emerges victorious in a game, resulting in an equal number of victories for multiple candidates. In this particular situation, it is necessary to establish a metric in order to resolve the tie.

## 3. Proposed Modifications

The localization ability of a method is reflected by the benchmark for long-term visual localization [21] based on the percentage of query images localized within *X*m and $Y^{\circ }$ of their ground truth pose. For most of the benchmark datasets, three intervals are defined as follows for measuring the pose accuracy under different thresholds: fine-precision (0.25 m, 2${}^{\circ }$), medium-precision (0.5 m, 5${}^{\circ }$), coarse-precision (5 m, 10${}^{\circ }$).

Precisely in view of the differences between the above two categories of datasets mentioned in Section 2.2, current visual localization challenges are evaluated separately for autonomous vehicles and handheld devices.

While the current benchmark is doing a great job handling these two challenges, our experience with its usage has shown that there are some areas we can improve. Firstly, the evaluation method is unable to completely eliminate human intervention. It is not feasible to determine whether the localization results of uploads with a uniform format have been artificially corrected or supplemented. In the case of autonomous vehicle datasets, obtaining extrinsic parameters of camera rigs or sequence information between images is relatively straightforward from the datasets. Consequently, this vulnerability may result in an unfair comparison between methods utilizing this correlation and pure visual localization methods that operate on individual images. Secondly, the ranking method occasionally produces ties, resulting in some entries appearing to have a higher rank than others, despite being ranked the same according to the ranking method.

As a means of evaluating numerous mainstream methods, the benchmark must possess objectivity and impartiality in order to effectively demonstrate the localization capabilities of all methods. This will ultimately contribute to the advancement of long-term visual localization. In light of this, we present our proposed modifications.

### 3.1. Evaluation Modification

As previously stated, the existing benchmark evaluation method relies on the percentage of query images successfully localized within the three defined thresholds. However, it is important to note that this method primarily assesses the localization results of handheld devices. In the case of autonomous vehicles, the objective of visual localization is to ascertain the precise position and orientation of the vehicle itself within the environment. Hence, we assert that the evaluation process should incorporate the localization results obtained from multiple cameras installed on the vehicle. In essence, the localization outcomes of these multiple cameras ought to be consolidated into a single vehicle pose to accurately calculate the overall accuracy, rather than being assessed individually.

This modification is absolutely necessary, since datasets with numerous cameras are frequently utilized and extrinsic camera calibration between the cameras used in the dataset can also be retrieved through given extrinsic files (e.g., RobotCar Seasons dataset [21,33]) or 3D models created from the reference images (e.g., CMU-Seasons dataset [21,32]). With the help of the extrinsic camera calibration, if one image taken at a specific timestamp is localized, then the pose for all images of the rig [37] (even if they were not successfully localized) can be estimated using the extrinsic camera parameters.

However, when dealing with datasets such as CMU-Seasons that lack intrinsic camera calibration, the utilization of the rig constraint becomes challenging. Submitters are left with no choice but to manually extract the rig information from the reference 3D model, as demonstrated in our own experiment in Section 4.2. Unfortunately, this manual extraction introduces variations due to different methods employed. Hence, we recommend implementing a standardized compensation process across the benchmark to ensure consistent vehicle pose acquisition.

Moreover, given the presence of query image sequences for autonomous vehicles, it becomes possible to estimate the pose of certain fail-localized images within these sequences through interpolation between the two nearest successfully localized images. Concerning the interpolation technique, linear interpolation stands as the most straightforward approach (as demonstrated in our actual experiment in Section 4.2). However, one could contemplate more sophisticated iterations that take into consideration timestamps, vehicle speed, or employ visual odometry for interpolation purposes. While these advanced interpolation methods may have a noticeable impact on the evaluation results, we do not want researchers’ attention to be diverted to these non-essential factors.

In theory, if submitters refrain from utilizing any interpolation methods, the outcomes will be reasonably equitable. However, in practice, this variable becomes uncontrollable. Consequently, we believe that the benchmark ought to execute a standardized and sufficiently precise interpolation incorporating sequence information as a preliminary step for computing the percentage results of all submitted methods. This will effectively eliminate the need for private interpolation and encourage a concentrated emphasis on the localization algorithm itself.

Regarding the specific modifications to the current evaluation method, it is important to note that they are not restricted to particular steps. The primary objective is to establish a standardized approach at the benchmark level, thereby mitigating the impact of human intervention on the evaluation process. For the convenience of illustration and explanation, the preprocessing operations adopted in this article are listed, consisting of two parts: rig operation and seq operation.

During the rig operation, the fixed extrinsic camera calibration data are first obtained from the dataset. Next, the vehicle position is defined as the pose of the left camera in the camera rig, and all the individual camera poses in the submitted results are compensated to it. This approach enables the determination of all the vehicle positions along the driving path, provided that at least one camera mounted on the vehicle is successfully located at that location.

During the seq operation, we iterate over the results of the rig operation to find the vehicle locations that have not been successfully located. Subsequently, we proceed to interpolate these identified locations with the sequence information in close proximity. Our interpolation radius ranges from small to large, indicating a prioritization of known position information closest to the unknown position. In the process of interpolation, we prioritize the use of known positions at both ends to predict the unknown position in the middle, and then consider using successive known positions to predict the next unknown position.

The impact of the above two operations using rig and sequence information on the evaluation results can be seen in Section 4.1 and Section 4.2. It is our firm belief that an exemplary benchmark should thoroughly assess the original outcomes of the localization algorithm, with minimal intervention from manual sources.

### 3.2. Ranking Modification

Since most of the datasets designed for long-term localization contain multiple conditions such as illumination (day/night), weather (sunny/rain/snow), and seasons (summer/winter), the localization results in each condition are also evaluated separately. This brings difficulties to comprehensive ranking. We studied the Schultze method employed by Sattler’s benchmark [21] and found that due to the limited number of evaluation conditions involved in some datasets, the ranking results may be partially tied due to the same number of leading conditions, as shown in Table 4, which may affect the ranking of the leaderboard, especially may cause disputes between the top few.

In the event of a deadlock, the Schulze Proportional Ranking method [38] effectively resolves the impasse by employing a random selection process to determine the victor among potential candidates. However, it is important to note that this approach may lack sufficient persuasiveness. Since the Schultze method is not a method specifically designed for visual localization, it treats the percentage of each threshold for each condition equally when calculating rankings. This processing method fails to demonstrate the robustness of a localization method under varying conditions, nor does it adequately quantify the proportion of fine-precision results achieved under specific conditions, while these factors hold significant importance in evaluating the efficacy of a localization method.

To this end, we introduce a secondary indicator CB, which combines the Intra-condition Balance (ICB) and Cross-condition Balance (CCB), to provide reasonable ranking criteria when the Schultze method has difficulty in choosing in a tie situation. Instead of relying on alternative indicators such as median error or overall lowest percentage correct in all conditions, our proposed CB method has been specifically designed to align with the demands of visual localization and to comprehensively reflect the localization ability.

Our idea comes from the Resolvability [22] of the Schultze method. It basically says that usually there is a unique winner $S=\left\{a\right\}$, and when there is more than one winner, then $S=\left\{a_{i}\right\}$, in that way, for every alternative $a_{i}\in S$, it is sufficient to add a single ballot *w* so that alternative $a_{*}\in S$ becomes the unique winner.

The secondary indicator CB is defined as follows:

(3) $$\begin{array}{cc} CB & =ICB+CCB\\ ICB & =\min(\frac{fine}{coarse})\\ CCB & =\min(\frac{\min(fine)}{\max(fine)},\frac{\min(medium)}{\max(medium)},\frac{\min(coarse)}{\max(coarse)}) \end{array}$$

where coarse, medium and fine are three arrays composed of percentage results corresponding to (5 m, 10${}^{\circ }$), (0.5 m, 5${}^{\circ }$) and (0.25 m, 2${}^{\circ }$) under different conditions.

Apparently, ICB is positively correlated with the minimum percentage gap of different thresholds under the same condition, while CCB is positively correlated with the minimum percentage gap of different conditions under the same threshold.

With the relaxation of the threshold, the percentage results increase monotonically, while the overall goal of visual localization is to obtain a fine-precision pose, so we believe that the coarse-precision threshold percentage reflects the basic localization ability of an evaluated algorithm. On this basis, under the same condition, a more precise algorithm has a higher fine-precision threshold percentage. Meanwhile, under the same accuracy threshold, a more robust algorithm has a smaller percentage difference between different conditions. CB thus comprehensively reflects the precise localization capability and the robustness of a method within and between conditions.

The impact of the above secondary indicator on the ranking results can be seen in Section 4.3. It is worth emphasizing that this secondary indicator only works when the current ranking method is stuck in a tie, and therefore does not affect the existing ranking of most algorithms. But, it addresses a potential weakness of the current benchmark and enhances its completeness and persuasiveness.

## 4. Experiments

This section presents a series of experiments conducted using both simulated and real data to validate the deficiencies of the benchmark evaluation and ranking methods previously proposed. It aims to demonstrate the improvements achieved by implementing our proposed modifications.

### 4.1. Simulation Experiment for Evaluation

We build two meticulously crafted simulation datasets for the purpose of localization testing. Each dataset comprises 200 reference positions derived from 100 acquisition points, replicating the scenario of two front-facing cameras installed on a vehicle. These cameras are positioned at a 45-degree angle to the left or right side of the vehicle, as demonstrated in the CMU-Seasons dataset [21,32].

**Figure 2:**
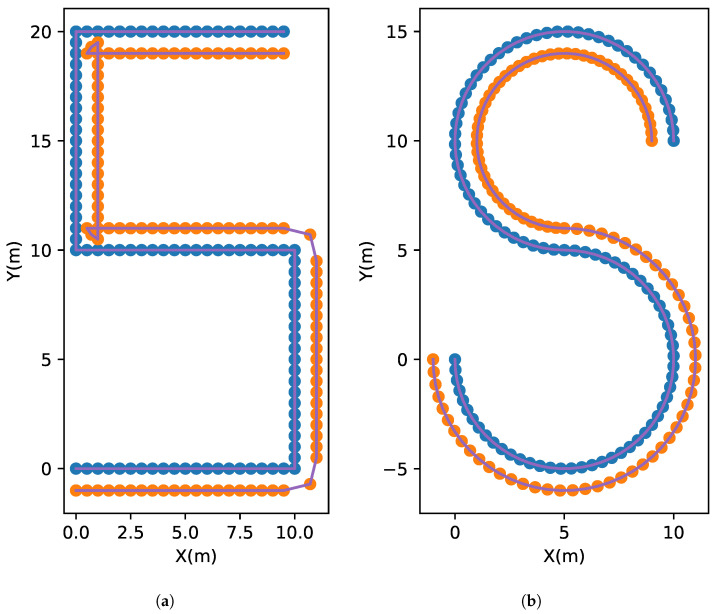
The real trajectory of the Straight (**a**) and Curved (**b**) routes. The blue points record the movement of the camera on the left side of the vehicle, while the orange points record the movement of the camera on the right side. The two cameras on the vehicle are positioned a meter apart.

The primary objective of these datasets is to accurately simulate the localization process during the movement of a vehicle along both straight and curved routes. In the case of the straight route, the true trajectory follows an “S”-shaped path, consisting of five consecutive straight lines, each measuring 10 m in length. These lines are vertically connected to one another, as depicted in the left half of Figure 2.

On the other hand, the curved route resembles an “S”-shaped path formed by two three-quarters circles with a radius of 5 m. As illustrated in the right half of Figure 2, these circles’ ends are joined to form a continuous path.

It should be noted that the path settings of the two simulation datasets are applied to the trajectory of the left camera. According to rig information, the right camera of the vehicle automatically generates its own trajectory based on the left camera’s known trajectory. The route discrepancies between the cameras on both sides are highlighted by this configuration.

For the purpose of clarity, we proceed by establishing the following assumptions:

- Each camera rises equally 1.5 m above the ground.
- All acquisition points are evenly distributed according to the camera on the left to conform to the image acquisition method of most data sets. As a result, the distance between every two connected blue points in the left half of Figure 2 is 0.5 m, and every two connected blue dots in the right half of Figure 2 correspond to one-fiftieth of the three-quarters of the central angle.
- The localization success rate of the simulated query images in the tested method is 70%.
- The orientation error of all simulation query images can be disregarded, thus the determination of the localization result’s interval is solely contingent upon the position error.

**Figure 3:**
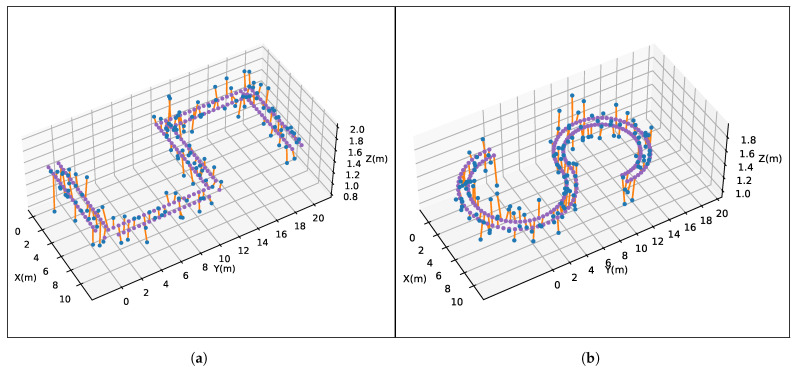
Distribution of basic data. (**a**) Straight route; (**b**) Curved route. The distribution of the basic localization results is shown by the blue points, while the purple points depict the actual trajectory. An orange line segment connects each blue point to its corresponding real position.

The localization results of two simulation experiments are generated by adding Gaussian noise with a mean value of μ=0 and a standard deviation of σ=0.2 on the basis of the real trajectory in Figure 2. To create the basic test data, 30% of the localization results are randomly eliminated. Referring to Figure 3, we can see the distribution of basic data.

We, respectively, evaluate the localization results of the simulated method by the current benchmark evaluation method and by our improved method. Each experiment contains three modes: Basic, Basic_rig, and Basic_rig_seq, which separately represent the basic results of the simulation method, the supplemented results with extrinsic camera calibration information, and the supplemented results with both rig information and sequence interpolation.

The rig and seq supplementation are executed in accordance with the methodology outlined in Section 3.1. In our enhanced approach, the left camera’s pose is established as the vehicle’s pose. Consequently, the ultimate determination of the vehicle’s pose is contingent upon the following four scenarios:

- If both cameras of the rig are successfully localized, the pose of the right camera is compensated to the left. The average of their poses is then regarded as the vehicle’s pose.
- If only the left camera is successfully localized, its pose will be regarded as the pose of the vehicle.
- If only the right camera is successfully localized, the subsequent compensated result of the pose to the left is regarded as the vehicle’s pose.
- If no camera is successfully localized, the vehicle is regarded as fail-localized.

Table 1 shows the localization results of simulation experiments. We present the data on the proportion of queries that have been successfully localized within different distance and orientation thresholds and emphasize the most effective technique for each.

The table demonstrates that both the Straight route and the Curved route exhibit the same tendency. Under each precision threshold in the current evaluation approach, rig operation and seq operation can significantly increase the localization algorithm’s accuracy. The rig operations of the two simulation experiments cannot, however, affect the basic results after substituting the improved method for the original one. Meanwhile, the improvement in the final results is much lessened even with the inclusion of seq operations (7.7% vs. 53.7% improvement for fine-precision threshold on Straight Route and 12.2% vs. 58.3% improvement for that on Curved route).

Results show that such a modification can entirely suppress the rig operation’s influence and lessen the seq operation’s level of influence.

### 4.2. Actual Experiment for Evaluation

To authenticate the influence of rig and seq supplementation on the localization results of real datasets, we conduct the ensuing experiment by utilizing the results of a prior study [20] on the CMU-Seasons dataset [21,32].

For the rig supplementation, it should be noted that the CMU-Seasons dataset [21,32] does not directly offer extrinsic parameters between the two cameras mounted on the vehicle. However, we are able to derive the real pose of the reference images from the 3D model provided within the dataset. Then, the average extrinsic camera parameters can be calculated with the advantage of the true pose of the paired reference images. Exploiting the synchronous capture of query images with a rig consisting of two cameras, we are able to detect the missing poses for all cameras that failed to localize, provided that the other camera in the rig, associated with those cameras, has been successfully localized.

For the seq supplementation, on the basis of the known poses, we look for fail-localized images and estimate their poses using linear interpolation between the two nearest successfully localized images.

Table 2 presents the benchmark evaluation results of UniGAN(RGBS) + NV + SP proposed by [20] and that of our supplementations. (It is worth mentioning that in the original UniGAN(RGBS) + NV + SP, the pose of a fail-localized image is approximated by the pose of the reference image that is most similar to it.) Similarly, the optimal results for each precision threshold are shown in bold.

The percentage results augmented by rig and seq options significantly outperform the basic results, as shown in the table, in practically all scenarios and across all thresholds. As a result, even without any optimization to the localization algorithm, we can significantly improve the evaluation results, especially for fine-precision percentages and the most difficult park scene that is most susceptible to appearance changes in the CMU-Seasons dataset [21,32]. This emphasizes the importance of consistent benchmark-level preprocessing.

### 4.3. Experiment for Ranking

The experiment in this section aims to validate the efficacy of our ranking improvement method in making a distinction where the Schultze method yields tied results. Within the benchmark leaderboard, each threshold for every condition corresponds to a distinct “voter” as per the Schultze method. For instance, one such voter is denoted as “fine-precision for night images” in the Aachen Day-Night dataset [21,29], while another is denoted as “medium-precision for day images”, and so on.

Unlike most electoral systems, as an evaluation system for visual localization algorithms, the number of “voters” is usually less than the number of “candidates”. This configuration increases the likelihood of encountering a deadlock situation, particularly in cases where the dataset portrays a limited number of scenes or when different methods yield equal percentages.

We use the data from the leaderboard of the Aachen Day-Night v1.1 dataset [21,29,39] on the benchmark. In order to enhance readability, we substitute the name of each method with a corresponding number. For specific information, please refer to Table 3 where the order of entries aligns with that on the benchmark.

In the first experiment, we employ the Schultze method on the ranking data in Table 3, the ranking results obtained are shown in Table 4, with four ties that can be seen in the 3rd, 10th, 12th, 14th place. Analyzing the ranking data of the methods in these deadlocks, we will find that they are either exactly the same or have the same number of votes among the six “voters”.

To break the ties, another ballot that combines the Intra-condition Balance (ICB) and Cross-condition Balance (CCB) is added. As long as this ballot has a strict partial order, the ties will be broken, according to the resolvability criterion of the Schultze method.

Take the tie of Method 3 and Method 4 in the third place in Table 4 as an example, the secondary indicator CB can be calculated for both methods following Formula (3).

For Method 3:

(4) $$\begin{array}{cc} ICB_{3} & =\min(\frac{90.0}{99.5},\frac{72.3}{97.9})=0.739\\ CCB_{3} & =\min(\frac{\min(90.0,72.3)}{\max(90.0,72.3)},\frac{\min(96.2,86.4)}{\max(96.2,86.4)},\frac{\min(99.5,97.9)}{\max(99.5,97.9)})=0.803\\ CB_{3} & =ICB_{3}+CCB_{3}=1.542 \end{array}$$

For Method 4:

(5) $$\begin{array}{cc} ICB_{4} & =\min(\frac{88.8}{99.0},\frac{74.3}{98.4})=0.755\\ CCB_{4} & =\min(\frac{\min(88.8,74.3)}{\max(88.8,74.3)},\frac{\min(95.4,90.6)}{\max(95.4,90.6)},\frac{\min(99.0,98.4)}{\max(99.0,98.4)})=0.837\\ CB_{4} & =ICB_{4}+CCB_{4}=1.592 \end{array}$$

Then, the seventh “voter” will come forward and say that he prefers Method 4, which determines the final order in the tie. Table 5 shows the ranking results of our improved method.

Except for the case where the data of Method 10 and Method 11 are exactly the same, our method produces a strict partial order of all methods. Compared with the original ranking in Table 3, the updated results exchange the ranking of Method 3 and 4, and clearly provide the basis for Method 4 to be superior to Method 3, which is more convincing.

## 5. Results

### 5.1. Simulation Experiment for Evaluation

The objective of this simulation experiment is to replicate the impact of rig operation and seq operation on the evaluation results of a specific localization method, and to substantiate, to a certain degree, whether the influence of these two operations is mitigated after implementing the proposed enhancement method. Due to the unavailability of the real benchmark’s test datasets, we are limited to utilizing two common path patterns, namely straight line and curve, to observe the implementation specifics of these two operations. Although there are evident disparities between the simulated scenarios and the actual environment, encompassing path morphology, distribution of simulated data, and the success rate of the localization method, the experimental findings adequately reflect the issue at hand. Specifically, operations resembling rig and seq have a significant impact on the localization results of the method, and, to some extent, this impact may even surpass the accuracy improvement achieved through the overall framework optimization of the algorithm. By adopting the proposed enhancements, the benchmark will experience a reduction in the magnitude of this impact.

### 5.2. Actual Experiment for Evaluation

The objective of this actual dataset experiment is to authenticate the dependability of the aforementioned simulation experiment. It is hard to ascertain if other submission techniques employ rig and seq operations, and it is not feasible to alter the assessment algorithm of the benchmark [21]. As a result, we are unable to conduct a comprehensive comparison of the localization results for all submission methods uniformly.

All we can do is perform reverse validation using the intervention methods mentioned above. Without incorporating our modifications into the existing benchmark, if human intervention is employed to complement the original results generated by the localization algorithm—specifically, using rig information to supplement the individual pose of certain fail-localized cameras, and subsequently using seq information to further complete the trajectory of each individual camera—what will be the extent of its impact on the evaluation results? The experiment results indicate that, at least in the context of our prior research [20], after introducing the two intervention operations, the evaluation outcomes in each subdivision of the CMU dataset have considerably improved, as depicted in Table 2. This underscores the indispensability of our modifications.

### 5.3. Experiment for Ranking

The objective of this ranking experiment is to replicate a potential deadlock situation and assess whether the inclusion of new indicators can yield logical ranking outcomes to resolve the deadlock dilemma. In order to accomplish this, the experimental data employed is sourced directly from the leaderboard of the Aachen Day-Night v1.1 dataset [21,29,39] on the benchmark. Although the leaderboard has a strict partial order as shown in Table 3, our analysis utilizing the Schultze method reveals the existence of several pairs of deadlock dilemmas. The benchmark does not specify the rationale behind the current strictly partial order results. Furthermore, according to the Resolvability [22] of the Schultze method, the default approach to breaking the equilibrium is random, which is evidently not rational. Following our intervention with secondary indices to address the local dilemma, our revised ranking results demonstrate a reversal of positions 3–4 and 14–15, when sufficient justification was present. This revised approach is evidently more logical than the original scheme of randomly breaking ties.

## 6. Discussion

### 6.1. Necessity

Admittedly, the amendments mentioned in this article make the benchmark more complex and unintuitive, and the rig and seq operations involved are more specific to autonomous vehicles. For datasets obtained from a solitary camera and datasets lacking sequence information, the consideration of the Canonical Reference setting is unnecessary. The current evaluation methods utilized in the benchmark are deemed satisfactory for these cases.

Furthermore, substituting camera rig localization results for multiple camera localization results does result in a reduction in evaluation sensitivity. This is evident in the fact that, at a specific location, the localization results are deemed successful as long as one camera in the rig is successfully located, regardless of any other camera localization failures. This makes it impossible to highlight some methods with a high success rate of individual localization. For this reduction, our view is that, since the existing benchmark has separated the evaluation into challenges for autonomous vehicles and handheld devices, the focus of autonomous vehicle localization should be on determining the posture of the vehicle itself, rather than the individual cameras.

Given the rapid advancements in autonomous driving and SLAM technology, visual localization is now encountering numerous instances of multi-sensor fusion and serialized images in real-world scenarios. Consequently, it becomes imperative to establish standardized evaluation criteria for localizing this specific subset of data. Our modification presents an additional dimension for evaluating datasets containing camera rigs and serialization information while maintaining the practical significance of the localization problem faced by autonomous vehicles. Such standardization will foster a fair and equitable environment for algorithmic competitions, ultimately leading to the emergence of more refined localization techniques that closely align with practical applications.

### 6.2. Influence

Changes related to benchmarks are potentially important as the benchmark is widely adopted for structure from motion, data association, and 6-DoF tracking/relocalization, etc. It may help to improve the benchmark to better measure the quality of a proposed matching/localization method. Therefore, it should also be treated with greater caution.

The inspiration for this endeavor was derived from our meticulous examination of prior work. Upon establishing a comprehensive framework for visual localization algorithms, we discovered that actions such as rigging and sequencing can significantly impact the algorithm’s performance on the benchmark. Although this influence may not dramatically alter the performance of the localization algorithm, it possesses sufficient potency to distinguish it from algorithms of comparable levels. Consequently, this phenomenon may impede researchers from identifying avenues that harbor genuine potential for groundbreaking advancements. It is precisely due to this rationale that we believe that only through the uniform treatment of marginal operations at the benchmark level can we genuinely ensure that researchers’ focus remains directed towards the development and exploration of algorithmic subjects.

The modification scheme proposed in this paper may not be flawless. For instance, during the compensation of the pose of multiple cameras to a pre-defined reference system of the vehicle, the camera extrinsic parameters utilized are obtained from the published data of the dataset, which typically contains an error. Consequently, the cameras apart from the canonical camera may exhibit additional noise during evaluation. How to avoid this effect might be worthy of future research.

## 7. Conclusions

In this paper, we present a series of proposed modifications to enhance the current benchmark for long-term visual localization. To accurately assess the localization ability of a submitted method on datasets comprising images captured by multiple cameras mounted on the vehicle, we suggest employing the compensated vehicle pose as an evaluation metric. Additionally, for datasets containing sequence information, we suggest performing a preprocessing step of standardized interpolation for a submitted method.

Moreover, we introduce an indicator based on existing data that provides a comprehensive reflection of a localization method’s cross-condition robustness and intra-condition fine-precision ratio. This indicator serves to mitigate any potential confusion arising from ties during ranking calculations.

The extensive experiments performed on both simulated and actual datasets confirm the deficiencies we discovered, thus demonstrating the necessity as well as the effectiveness of our modifications.

## Figures and Tables

**Table 1:** Localization results of simulation experiments.

		Current Method	Improved Method
	distance [m]	0.25/0.50/5.0	0.25/0.50/5.0
	orient. [deg]	2/5/10	2/5/10
Straight Route	Basic	20.5/62.5/70.0	39.0/86.0/93.0
	Basic_rig	27.5/82.0/93.0	39.0/86.0/93.0
	Basic_rig_seq	**31.5**/**88.0**/**100.0**	**42.0**/**92.0**/**100.0**
Curved Route	Basic	24.0/65.5/70.0	49.0/86.0/91.0
	Basic_rig	32.5/84.0/91.0	49.0/86.0/91.0
	Basic_rig_seq	**38.0**/**92.5**/**100.0**	**55.0**/**95.0**/**100.0**

* The most efficient methods for each threshold are highlighted in bold.

**Table 2:** Localization results of actual experiments.

CMU-Seasons						
	Urban	Suburban	Park	Overcast	Sunny	
distance [m]	0.25/0.50/5.0	0.25/0.50/5.0	0.25/0.50/5.0	0.25/0.50/5.0	0.25/0.50/5.0	
orient. [deg]	2/5/10	2/5/10	2/5/10	2/5/10	2/5/10	
UniGAN(RGBS) + NV + SP [20]	92.4/95.0/98.0	75.9/82.1/91.0	56.8/65.1/81.7	78.6/84.0/**92.4**	69.0/74.5/87.2	
UniGAN(RGBS) + NV + SP_rig	93.3/95.8/98.0	81.4/88.6/94.8	65.3/74.3/82.8	80.9/86.2/90.6	76.9/83.3/89.5	
UniGAN(RGBS) + NV + SP_rig_seq	**93.4**/**96.1**/**98.4**	**81.6**/**89.2**/**96.1**	**66.5**/**77.4**/**89.7**	**81.1**/**86.7**/92.3	**78.2**/**86.5**/**96.2**	
	foliage	mixed foliage	no foliage	low sun	cloudy	snow
distance [m]	0.25/0.50/5.0	0.25/0.50/5.0	0.25/0.50/5.0	0.25/0.50/5.0	0.25/0.50/5.0	0.25/0.50/5.0
orient. [deg]	2/5/10	2/5/10	2/5/10	2/5/10	2/5/10	2/5/10
UniGAN(RGBS) + NV + SP [20]	71.7/76.9/88.4	75.2/81.5/90.8	82.9/87.9/93.4	75.4/81.8/90.4	80.9/85.2/92.6	74.8/81.8/89.3
UniGAN(RGBS) + NV + SP_rig	78.9/84.8/90.2	78.1/84.6/90.5	85.5/90.9/95.7	79.2/86.0/91.9	84.9/89.5/93.6	80.0/87.8/94.1
UniGAN(RGBS) + NV + SP_rig_seq	**79.9**/**87.3**/**95.6**	**78.3**/**85.2**/**92.1**	**85.7**/**91.3**/**96.5**	**79.4**/**86.6**/**93.7**	**85.2**/**90.4**/**95.1**	**80.4**/**88.6**/**95.8**

* The most efficient methods for each threshold are highlighted in bold.

**Table 3:** Ranking data from the leaderboard of the Aachen Day-Night v1.1 dataset.

Method	Day	Night
distance [m]	0.25/0.50/5.0	0.25/0.50/5.0
orient. [deg]	2/5/10	2/5/10
Method 1	90.9/96.7/99.5	78.5/91.1/98.4
Method 2	89.8/96.1/99.4	77.0/90.6/100.0
Method 3	90.0/96.2/99.5	72.3/86.4/97.9
Method 4	88.8/95.4/99.0	74.3/90.6/98.4
Method 5	86.0/94.8/98.8	72.3/88.5/99.0
Method 6	87.1/94.7/98.3	74.3/86.9/97.4
Method 7	0.0/0.0/0.0	73.3/88.0/98.4
Method 8	0.0/0.0/0.0	69.1/87.4/98.4
Method 9	0.0/0.0/0.0	73.3/86.9/97.9
Method 10	0.0/0.0/0.0	71.2/86.9/97.9
Method 11	0.0/0.0/0.0	71.2/86.9/97.9
Method 12	0.0/0.0/0.0	72.3/86.4/97.4
Method 13	0.0/0.0/0.0	72.3/85.3/97.9
Method 14	0.0/0.0/0.0	67.5/85.9/97.4
Method 15	76.5/85.9/94.1	34.6/41.4/49.7

**Table 4:** Ranking results of the Schultze method.

Rank	Method
1	Method 1
2	Method 2
3	Method 3, Method 4
5	Method 5
6	Method 6
7	Method 7
8	Method 8
9	Method 9
10	Method 10, Method 11
12	Method 12, Method 13
14	Method 14, Method 15

**Table 5:** Ranking results of the improved Schultze method.

Rank	Method
1	Method 1
2	Method 2
3	Method 4
4	Method 3
5	Method 5
6	Method 6
7	Method 7
8	Method 8
9	Method 9
10	Method 10, Method 11
12	Method 12
13	Method 13
14	Method 15
15	Method 14

## Data Availability

Data available in a publicly accessible repository that does not issue DOIs. Publicly available datasets were analyzed in this study. This data can be found here: https://www.visuallocalization.net/.

## Abbreviations

The following abbreviations are used in this manuscript:

6-DOF	6-Degree-of-Freedom
SLAM	Simultaneous Localization and Mapping
CB	Condition Balance
ICB	Intra-condition Balance
CCB	Cross-condition Balance
